# Time-use and environmental determinants of dropout from organized youth football and tennis

**DOI:** 10.1186/s12889-018-5919-2

**Published:** 2018-08-16

**Authors:** Ineke Deelen, Dick Ettema, Carlijn B. M. Kamphuis

**Affiliations:** Department of Human Geography and Spatial Planning, Faculty of Geosciences, Utrecht University, Princetonlaan 8A, 3584 CB Utrecht, The Netherlands

**Keywords:** Sports participation, Dropout, Sports clubs, Adolescents, Time-use, Environment, Football, Tennis

## Abstract

**Background:**

Many adolescents drop out of organized sports. Lack of motivation and competing priorities are known as important reasons for dropout. However, time use factors as well as environmental determinants have been largely neglected in the current literature on dropout from youth sports. The aim of this study is to investigate how (changes in) time use and characteristics of the physical environment determine dropout from football and tennis among adolescents.

**Methods:**

Data on time use and background characteristics were collected through online surveys in 2015 and 2016 among adolescents aged 13–21 (*N* = 2555), including both the dropped outs and those who still continued membership of their football or tennis clubs. Physical environmental determinants (travel distance to the sports club, and neighbourhood density) were measured objectively. Binary logistic regression analyses were carried out for football and tennis separately to examine the associations between time use (time spent on various activities and changes related to the school and job situation), and environmental factors on the probability of dropping out from sports.

**Results:**

Time spent on sports outside the context of the sports club, and time spent on social or voluntary activities at the sports club was positively associated with continuing being football and tennis members. Tennis players who changed schools or participated in two sports at the same time had a higher probability of dropping out, whereas tennis players who travelled greater distances from home to the tennis club were less likely to drop out.

**Conclusions:**

Determinants of dropout differed between football and tennis. However, time use variables were important predictors of dropout from football as well as tennis, whereas environmental determinants hardly contributed to the prediction of dropout. To keep youths involved in organized sports, this study recommends that sports professionals should: 1) offer flexibility in training and competition schedules, 2) stimulate participation in social activities and voluntary work at the sports club, 3) pay special attention to their needs and preferences, and 4) encourage possibilities to practice and play sports outside of regular training hours, for instance at the sports club or at playgrounds or parks in the neighbourhood.

## Background

Ample evidence exists that children and adolescents participating in sports improve their physical and mental health [1, 2]. Particularly, participation in organized sports has been found to be associated with greater psychological and social benefits in children and adolescents compared to individual, unorganized types of sports [1]. Because physical activity levels decline and sedentary lifestyles increase during adolescence [3, 4], particularly among girls [5–7], policies encourage a rise in sports participation among youths. Previous studies have shown that youths that do participate in organized sports show higher overall leisure time physical activity levels than youths not participating in sports [8–10]. Furthermore, studies also show that sports participation and other vigorous types of physical activity have positive health effects, independent of overall physical activity levels [11, 12].

In many European countries, sports clubs are the most common setting for sports participation among youths [13]. About 74% of all Dutch children aged 6–11, and 58% of adolescents aged 12–20 participate in sports at least once a week as member of a sports club [14]. However, sports membership rates decline sharply after the age of 14 [15] and this pattern is observed internationally [5, 16, 17]. Dropout rates from at least one type of organized sports have been estimated at 30 and 35% yearly among Canadian and Australian youths aged 5–15 and children aged 10, respectively [18, 19]. Møllerløkken, Lorås & Vorland Pedersen [20] reported in their international review that the annual weighted mean dropout rate in football was 23.9% among youths aged 10–19. However, dropout rates were higher for girls (26.8%) than for boys (21.4%).

Although participation in sports clubs is traditionally high among youths in the Netherlands, both the largest (football) and second largest organized sports (tennis) have to deal with significant declines of youth members. Data of Dutch sports federations showed that 31% of girls and 26% of boys aged 13–21 dropped out from football during the 2015/2016 season (total dropout rate 27%), in contrast to an increase of only 12% new youth members in this age group (‘dropped ins’). For tennis, dropout rates for girls and boys aged 13–21 were both 28%, whereas the annual drop-in rate was 11% in 2015/2016.

To reduce dropout from organized sports during adolescence, a better understanding of the determinants of dropout is required. In the literature, dropout or youth sport attrition has been addressed from various theoretical perspectives, focussing on different determinants. Particularly, intrapersonal determinants have been studied extensively [18, 21]. Studies showed that important intrapersonal determinants associated with dropout during late childhood and adolescence were: biological factors that include physical maturation and injuries; and socio-demographic determinants such as sex (girls), age, and lower socioeconomic household status [18, 19, 21–26]. Much evidence has been established for psychological determinants of dropout, which include lack of (intrinsic or a high level of self-determined) motivation and lack of (perception for) competence, autonomy and relatedness; concepts derived from the Self Determination Theory (SDT) [18, 27–29]. Another main theoretical approach used to understand the role motivation plays in understanding sports participation is the Achievement Goal Theory (AGT) [30]. The AGT distinguishes between two types of goal orientations, or personal definitions of success: task orientation and ego orientation. Whereas task orientated individuals focus on maximal effort and personal improvement, ego orientated individuals believe that winning and favourable outcomes are markers of success in sports [31]. Based on the AGT framework, several studies revealed that a task orientated personal goal orientation, and a task-orientated training climate, were associated with persistence in sports. This is in contrast to ego-orientation and the perception of an ego-orientated motivational climate, which were associated with dropout from youth sports [27, 31, 32]. Both theories AGT and SDT are complementary and studies have shown evidence for the adaptive role of high task orientation in promoting self-determination in sport. Task orientation was related to a higher level of self-determined or intrinsic motivation, and ego orientation with controlled forms of motivation [33]. Both theories emphasize the important (mediating) role of competence and predict that high perceived competence will sustain and increase one’s motivation to sport [18, 33, 34]. For instance, sports participants with a high task orientation are less likely to feel incompetent in sports than those with a high ego orientation [35]. Other studies highlight the importance of interpersonal determinants of dropout from organized youth sports, such as the social environment (e.g., lack of support from significant others such as parents, coaches and peers), as well as developmental factors (e.g., early diversification, later specialization in sports training, training patterns and being older than the rest of the team, which is known as the relative age effect) [19, 32, 36, 37].

While intrapersonal and interpersonal determinants of dropout from youth sports have been studied extensively, other determinants are largely neglected in the literature. In this study, we identified the role of time use and change in time use, as well as factors related to the physical environment in determining dropout from football and tennis among adolescents to fill this gap. Time pressure and competing life priorities are often mentioned as reasons for adolescents’ dropout from sports [21–23]. However, little is known about how time spent on different activities at certain locations is related to dropout. Insights from time-geography might add to the understanding of associations between time use and dropout from sports. From a time-geographical perspective, in a certain ‘time window’ only a limited set of places can be visited. Time use and participation in activities at a specific geographical location at a specific time are subject to constraints at biological, intrapersonal, interpersonal and institutional levels [38]. For example, some adolescents might experience constraints to continue participating in organized sports with fixed training and competition schedules, due to an increase in time spent in schools or studies, or the start of a job. Logically, such constraints are more severe if work or study is combined with multiple leisure activities, such as engaging in multiple sports and social activities. In addition, change of schools (from elementary to secondary education or from secondary to higher education), might increase travel time and study load, which might influence dropout from sports. A similar association has been found for declines in physical activity among adolescents who have changed schools, those who have started university or college or engaging in full-time work [39, 40].

Many studies investigating determinants of sports participation and physical activity built on socio ecological models and consider the effect of the physical environment on sports participation and physical activity [41, 42]. Various studies have demonstrated that the physical environment (e.g., distance to sports facilities, availability of sports facilities, green space, and neighbourhood characteristics such as neighbourhood density) is associated with an increase in participation in sports and physical activity [43, 44]. However, with regard to the effect of these factors on sports participation, the research findings are mixed and seem to apply to the youth living in low-socioeconomic areas or in rural communities [45–47]. Environmental characteristics that are modifiable are of special interest as they provide opportunities to develop environmental interventions (e.g., to improve the accessibility to sports facilities and sports clubs) that may prevent dropout from sports. However, little is known about how and which characteristics of the physical environment influence dropout from sports among adolescents. For example, a review on dropout from youth sports carried out by Balish et al. [18] shows only one environmental determinant, which correlates only weakly with dropout. Contrary to their expectations, Boiché & Sarrazin [48] found that adolescents who continued with sports reported longer travel distances to their sports activities than those who dropped out.

**Fig. 1:**
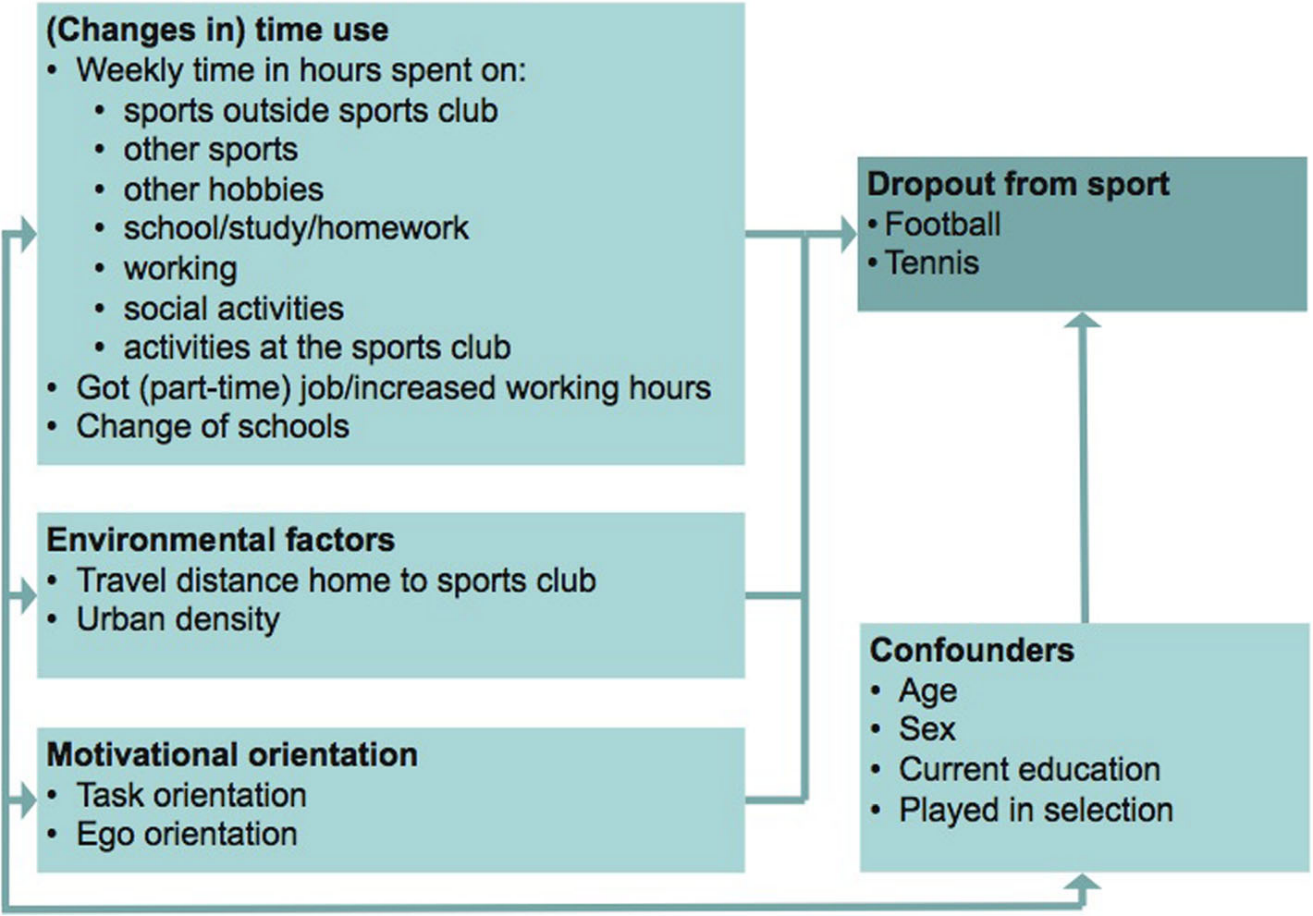
Conceptual framework

This present study explores the effects of time use and the physical environment on dropout from organized sports among youths, with a focus on football and tennis. First, we investigate how time use in competing activities (school, work, other sports, hobbies, social activities, and activities at the sports club), influences the probability of dropping out from organized football and tennis. This study focuses on a relatively broad age range (13–21) compared to other studies [18], in order to explore how important changes in the lives of adolescents that may affect time use, spatial setting and travel distance to the sports club, such as changing schools (from primary to secondary or from secondary to higher education) or entering the labour force are associated with dropout from sports. Moreover, we investigate the role of the physical environment, which includes distance to the sports facility and neighbourhood density, on the probability of dropping out. Finally, we investigate the relative importance of temporal and environmental determinants, compared to intrapersonal factors, such as task and ego orientation and socio demographic factors on dropout from sports. Figure 1 shows the conceptual framework of this study.

## Methods

### Design, setting and respondents

Data were collected via online surveys among adolescent football and tennis players, including both dropouts and those who continued with club membership. Dropouts were defined as those who ended their club membership over the past year. Data collection among dropouts and members of the Royal Dutch Football Federation (KNVB) took place in a year interval between May/June 2015 and June 2016. Moreover, data were collected between December 2015 and May 2016 among the dropouts and members of the Royal Dutch Lawn Tennis Federation (KNLTB). From each group 10,000 adolescents aged 13–21 years old were randomly selected from the membership registration databases. We focussed on youths aged 13–21 because of the potential differences in (changes in) time use and its’ spatial consequences in this life phase (changing schools, moving to another place due to studies, increased travel distance). The sports federations sent invitation letters for the survey via email. For those below 18 years old, written parental consent and adolescent assent were asked in the first question of the survey. In total 2566 respondents completed the surveys. Response rates varied from 10.1% for football members, 3.5% for football dropouts, 6% for tennis members to 6% for tennis dropouts (total response rate was 6.4%). Respondents with inaccurate socio-demographic data or inaccurate contact addresses (*N* = 11) were excluded from further analysis. Complete data of 2555 adolescents were available as shown in Table 1. A comparison of age and sex distributions between our study subsamples and the national membership and dropout data of the Dutch football and tennis unions, did not point towards a selection bias regarding age groups or sex. However, an exception is the sex distribution for football dropouts. In our study sample, 43% of the football dropouts were women whereas nationally 19% of the dropouts were women.

**Table 1:** Personal characteristics of football and tennis players and dropouts, time use, environmental, and motivational factors

	Total (*N* = 2555)	Football members (*N* = 1014)	Football dropouts (*N* = 346)	Tennis members (*N* = 602)	Tennis dropouts (*N* = 593)
*Socio-demographic factors*					
Age (%)					
13–16	52.4	61.4	37.6	44.9	53.3
17–21	47.6	38.6	62.4	55.1	46.7
*Mean (SD)*	16.5 (2.5)	16 (2.3)	17.5 (2.3)	17.1 (2.6)	16.5 (2.5)
Female (%)	39.1	19.7	43.1	59	49.7
Current education (%)					
No	3.3	3.6	1.4	4.8	2.4
Low	37.9	57.2	42.2	19.3	21.2
Middle	38.2	32.4	26.9	43.2	49.4
High	20.6	6.7	29.5	32.7	27
*Sports participation characteristics*					
Played in selection team (%)					
Yes	40.4	53	34.7	39.7	22.8
No/don’t know	59.6	47	65.3	60.3	77.2
Past sports frequency (tennis/football) (%)					
No or less than once a week	14.7	2	7.5	9.6	45.7
Once a week	17.2	5.6	14.7	24.6	30.9
Twice a week	30.6	25.8	68.8	29.6	17.5
> Twice a week	37.5	66.6	9	36.2	5.9
*Time use factors*					
Time use indicators *at this moment* (average hrs per week), *mean (SD)*					
Tennis/football outside sports club (hrs)	1.7 (2.4)	2.8 (2.6)	0.4 (1.3)	1.9 (2.4)	0.1 (0.5)
Other sports (hrs)	2.6 (2.8)	2.0 (2.4)	2.2 (2.7)	2.3 (2.5)	4.1 (3.3)
Other (fixed) hobbies (hrs)	1.5 (2.4)	1.4 (2.4)	1.6 (2.6)	1.5 (2.2)	1.7 (2.5)
School and homework/studying (hrs)	19.8 (13.9)	17.5 (13.5)	17.9 (13.8)	22.2 (13.9)	22.3 (13.9)
(Part-time) job (hrs)	7.6 (10.1)	7.6 (10.4)	10.5 (11.3)	7.5 (9.9)	6.2 (8.6)
Social activities (no of times)	2.6 (1.3)	2.6 (1.4)	2.5 (1.3)	2.4 (1.2)	2.6 (1.4)
Total amount of time spent on social and other activities^a^ at sports club per week *past season* (hrs)	1.3 (4.7)	1.2 (3.3)	0.4 (1.6)	2.1 (7.0)	1.0 (4.8)
Changes in job or school situation between membership and dropout time (dropouts)/ during the past year (members) (%)					
Got (part-time) job/increased work hrs	18.9	22.7	19.9	21.9	8.9
Changed schools/further education	26.2	21.4	23.4	22.1	40.1
*Environmental factors*					
Travel distance to sports club (km), *mean (SD)*	3.9 (11.2)	3.6 (9.0)	3.9 (14.0)	6.0 (16.2)	2.5 (4.5)
Neighbourhood density (%)					
Rural	30.3	32.3	36.4	27.4	26.3
Hardly – moderately urbanized	40.4	37.2	37.6	42.5	45.4
Strong – extremely urbanized	29.3	30.6	26	30.1	28.3
*Motivational factors*					
Motivational orientation for sports during membership, *mean (SD)*					
Task orientation	0.0 (1.0)	0.1 (1.0)	−0.2 (1.1)	0.1 (1.0)	−0.1 (0.9)
Ego orientation	0.0 (1.0)	0.0 (1.0)	−0.2 (1.0)	0.1 (1.0)	0.0 (0.9)

^a^Other activities at the sports club such as (assistant) trainer, bar keeper, committee/board member, arbitrator (only in football)

### Measures

The online survey aimed to collect data on sports participation characteristics, time-use, and recent changes in job, home or school situation, as well as task and ego orientation. Socio-demographic characteristics and contact addresses of the home and sports club of the participants were obtained via membership registration databases of the sports federations.

#### Outcome variable

The outcome variable (dropout) was defined as a decision to resign from sports membership over a year prior to the survey (dropout = 1, and member = 0).

#### Time use characteristics

Time use items included time (in hours) spent on: 1) football or tennis outside the sports club, 2) another type of sports than football or tennis, 3) regular hobbies or activities outside of home, 4) school and homework or study, 5) part-time or fulltime work, and 6) social activities (in number of times per week). These questions on time use are referred to the time spent on these activities at the moment the survey took place. Since the time use items were assessed as categorical variables with a large number of categories, we convert them into continuous scores of the average hours spent on activities. Time spent on the activities at the sports club, such as coaching, being member of the board or social activities was dichotomized into ‘yes’ and ‘no’. These items were referred to the previous year, when dropouts were still members of the sports club. Furthermore, changes in job or school situation (as dummy variables) included: 1) starting a (part-time) job and/or increase in working hours during the past year (yes/no), and 2) changing schools (from primary to secondary or secondary to higher education) or change of school location over the past year (yes/no).

#### Environmental factors

Environmental determinants included travel distance from home to the sports club and density of the residential neighbourhood. Travel distance was measured objectively, as the distance in metres from home location to the location of the sports club. Both locations were determined by Google’s Geolocation API based on their full addresses (street name and house number) [49]. Bicycle paths were used as the transport network because the majority of respondents cycled to the sports club (79.1%).

Neighbourhood density was based on the number of addresses within a radius of one square kilometre from the home location [50], and was aggregated to a 4-digit postal code level. Three categories of address density were distinguished: rural (< 500 addresses per km^2^), hardly to moderately urbanized (500–1.500 addresses per km^2^), and strongly to extremely urbanized (> 1.500 per km^2^).

#### Task and ego orientation

Task and ego orientations were measured by using the validated and reliable Dutch version of the Task and Ego Orientation in Sports Questionnaire (TEOSQ) [51]. Respondents were asked to indicate when they felt most successful in sports, by indicating to what extent they agreed with 7 items that reflected task orientation and 6 items that reflected ego orientation. Examples of such items are “I learned a new skill by trying very hard” (for task orientation), and “I could do better than my teammates” (for ego orientation). A 5-point Likert scale was used ranging from 1 (strongly disagree) to 5 (strongly agree). In this study, Cronbachs’ alpha levels for the task and ego orientation scales were 0.91 and 0.88, respectively, and 0.88 for the total scale (Table 2). Table 2 also shows the results of a Principal Component Analysis (PCA) for the items of the TEOSQ. Average PCA scores for the task and ego orientation were used as continuous measures for task and ego orientation.

**Table 2:** Principal components analysis on task and ego orientations for participation in football/tennis

Construct/item “I feel/felt successful as a tennis/football player when…”	Task orientation	Ego orientation	Total
Something I learned makes me want to go and practice more	0.81	0.21	
I learned something that was very fun to do	0.79	0.10	
I learned a new skill by trying very hard	0.78	0.17	
I did my very best	0.77	0.17	
I learned a new skill and it made me want to practice more	0.77	0.24	
I worked really hard	0.74	0.21	
A skill I learned really felt right	0.73	0.32	
I was the best	0.10	0.86	
The others couldn’t do as well as me	0.08	0.86	
I could do better than my teammates	0.25	0.81	
I contributed most to the victory	0.24	0.74	
I was the only one who could do the play or skill	0.40	0.63	
Others messed up and I didn’t	0.22	0.62	
Eigenvalues	6.18	2.11	
Explained variance, %	34.71	29.04	
Explained variance, cumulative %	34.71	63.75	
Cronbach’s alpha (based on standardized items)	0.91	0.88	0.88
Scale mean	3.71	3.122	3.41

#### Confounders

We controlled the following socio demographic characteristics: age, sex (13–16 and 17–21) and education. For education, we distinguished between four levels of the level of current education: 1) no education, 2) lower education (i.e. primary education, lower professional education), 2) middle education (i.e. intermediate and higher general education), and 3) higher education (professional education and university). Furthermore, we controlled whether the participants ever played in a selection team (yes or no), because the competitive level of sports participation might influence dropout [18].

### Statistical analysis

Descriptive analyses were carried out to determine respondents’ personal characteristics, time use characteristics, and environmental determinants. Subsequently, binary logistic regression analysis on the probability of dropping out of sports (dropout vs member as the outcome variable) was estimated to examine the effects of confounders (Model 1). Moreover, time use variables (Model 2), environmental factors (Model 3), and task and ego orientation (Model 4) were added for subsequent analysis. The analyses were performed for football and tennis separately, because descriptive results showed significant differences between football and tennis. All the analyses were carried out with SPSS version 24.0.

## Results

### Descriptive results

Table 1 shows socio-demographic, sports participation, time use and environmental characteristics of the study sample. Mean age was 16.5 (SD = 2.5) years, and 61.9% was male. Sex differences in types of sports and dropout status were found, with relatively large shares of male football members (80.3%) and female football dropouts (43.1%). Tennis members (32.7%) and dropouts (27%) were more frequently higher educated, compared to football members (6.7%) and dropouts (29.5%).

Time spent in schools and homework/studying consumed the highest time, especially in tennis members and tennis dropouts. Tennis dropouts most frequently changed schools (40.1%). Football members and football dropouts spent more time on work, and experienced a change in their work situation (e.g., started a new job, or increased work hours during the past year) more frequently. Tennis members travelled on average 6 km (SD = 16.2) from home to their tennis club, which was more than the average of the sample (3.9 km; SD = 11.2 km). In contrast, travel distance among tennis dropouts was relatively low (2.5 km; SD = 4.5 km).

### Multivariate analyses of football dropout

Results of binary logistic regression analysis on the probability of dropout from football (Table 3), showed that time use variables were important in the prediction of drop out of football: Nagelkerke R-squared was 0.21 in the baseline model with confounders, compared to 0.50, in the model when time use variables were added. Results showed that girls who played football were more likely to dropout than boys. Those with a higher level of education and those who played in a selection team were associated with lower odds on dropout. The second model showed that adolescents who spent more time on football *outside* the sports club, as well as those who spent more time on voluntary/social activities *at* the sports club, were less likely to drop out from organized football. Environmental factors (model 3) did not significantly explain the probability of dropout. The final model showed that both football players who were more task orientated, and those more ego orientated were less likely to drop out compared to those who were less motivated no matter their motivational orientation.

**Table 3:** Binary logistic regression on dropout (yes/no) in sports – FOOTBALL (*N* = 1360)

Outcome = Dropout vs Member	Model 1 (socio-demographic factors)			Model 2 (model 1 + time use factors)			Model 3 (model 2 + environmental factors)			Model 4 (model 3 + motivational orientation)		
	Exp (B)	95% CI	P	Exp (B)	95% CI	P	Exp (B)	95%	P	Exp (B)	95% CI	P
Constant	1.71		0.005	5.24		0	4.53		0	4.00		0
*Control variables*												
Age (17–22 = ref)												
13–16	0.48	0.36–0.64	0.000	0.90	0.62–1.32	0.597	0.90	0.61–1.31	0.597	0.90	0.61–1.33	0.598
Sex (male = ref)	2.41	1.81–3.20	0.000	1.73	1.23–2.43	0.002	1.71	1.22–2.41	0.002	1.78	1.26–2.51	0.001
Current education (high = ref)						0			0			0
No	0.13	0.05–0.34	0.000	0.08	0.03–0.27	0	0.08	0.03–0.26	0	0.07	0.02–0.24	0
Low	0.25	0.17–0.37	0.000	0.28	0.17–0.45	0	0.27	0.17–0.45	0	0.27	0.16–0.44	0
Middle	0.27	0.18–0.41	0.000	0.30	0.18–0.50	0	0.30	0.18–0.50	0	0.29	0.17–0.49	0
Played in selection (team) (no = ref)	0.46	0.35–0.61	0.000	0.68	0.48–0.95	0.023	0.67	0.48–0.94	0.022	0.70	0.50–0.99	0.041
*Time use factors*												
Time use indicators *at this moment* (average hrs per week)												
Tennis/football outside sports club				0.30	0.25–0.36	0	0.30	0.25–0.36	0	0.31	0.26–0.37	0
Other sports				1.03	0.97–1.10	0.387	1.03	0.97–1.10	0.387	1.04	0.97–1.11	0.254
Other (fixed) hobbies				1.04	0.98–1.11	0.206	1.04	0.98–1.11	0.21	1.04	0.98–1.11	0.205
School/study and homework				0.99	0.98–1.0	0.187	0.99	0.98–1.00	0.176	0.99	0.98–1.01	0.212
(Part-time) job				1.01	1.00–1.03	0.125	1.01	1.00–1.00	0.13	1.02	1.00–1.03	0.073
Social activities *(no of times)*				0.97	0.86–1.09	0.599	0.97	0.86–1.09	0.593	0.98	0.86–1.10	0.687
Time spent on side activities at sports club *past season* (hrs)				0.89	0.81–0.89	0.019	0.89	0.81–0.98	0.016	0.89	0.81–0.98	0.019
Changes in job/school situation *since past season*												
Got job/increased work hrs (no = ref)				0.70	0.46–1.06	0.089	0.69	0.46–1.05	0.083	0.67	0.44–1.02	0.064
Changed schools/further educ. (no = ref)				0.80	0.53–1.21	0.294	0.81	0.53–1.22	0.31	0.78	0.51–1.20	0.255
*Environmental factors*												
Travel distance to sports club (km)							1.00	0.99–1.01	0.878	1.00	0.99–1.01	0.832
Neighbourhood density (strong – extremely urbanized = ref)							1.23		0.42	1.19		0.484
Rural							1.29	0.82–1.85	0.314	1.27	0.80–1.80	0.393
Hardly – moderately urbanized							4.53	0.87–1.92	0.203	0.77	0.85–1.90	0.236
*Motivational factors*												
Task orientation										0.85	0.66–0.89	0
Ego orientation										4.00	0.73–1.00	0.047
-2 Log likelihood	1332.57			977.88			976.13			960.20		
Cox&Snell R^2^	0.14			0.34			0.34			0.35		
Nagelkerke R^2^	0.21			0.50			0.50			0.51		

### Multivariate analyses of tennis dropout

Table 4 presents the results of binary logistic regression analyses on the probability of dropout from tennis. The results demonstrated that girls and those who played in a selection team were less likely to drop out. The time use variables increased the explanatory power of the model (Nagelkerke R-squared) from 0.07 to 0.51. When time use variables were added to the model, education became significantly associated with tennis drop out: tennis players with a middle/intermediate level of education were more likely to quit tennis compared to those with a higher level of education. Tennis players who spent more time on tennis outside their sports clubs were less likely to drop out, whereas tennis players who spent more time on other sports than tennis, and on social activities, were more likely to drop out of tennis. Change of schools much more increased the odds of dropping out of tennis (odds ratio 2.96, 95% CI 2.04–4.28), whereas starting a (part-time) job or an increase in work hours decreased the likelihood of dropping out. Results of the third model showed that those who travelled larger distances to the tennis club were less likely to drop out. Lastly, both task and ego orientated tennis players were less likely to dropout. The significant effect of sex disappeared when time use and environmental factors were added to the model.

**Table 4:** Binary logistic regression on drop out (yes/no) in sports – TENNIS (*N* = 1195)

Outcome = Dropout vs Member	Model 1 (socio-demographic factors)			Model 2 (model 1 + time use factors)			Model 3 (model 2 + environmental factors)			Model 4 (model 3 + motivational orientation)		
	Exp (B)	95% CI	P	Exp (B)	95% CI	P	Exp (B)	95%	P	Exp (B)	95% CI	P
Constant	1.71		0.005	0.58		0.111	0.67		0.257	0.63		0.208
*Control variables*												
Age (17–21 = ref)												
13–16	1.14	0.83–1.56	0.424	0.96	0.64–1.45	0.852	0.96	0.64–1.45	0.853	0.95	0.63–1.43	0.808
Sex (male = ref)	0.70	0.55–0.89	0.003	0.74	0.54–1.00	0.049	0.75	0.55–1.01	0.061	0.74	0.55–1.02	0.063
Current education (high = ref)			0.165			0.039			0.107			0.093
No	0.54	0.27–1.08	0.080	0.91	0.37–2.23	0.837	0.86	0.35–2.11	0.74	0.83	0.34–2.04	0.682
Low	1.09	0.75–1.60	0.640	1.42	0.86–2.32	0.169	1.28	0.78–2.11	0.336	1.21	0.73–2.00	0.464
Middle	1.19	0.83–1.72	0.343	2.00	1.21–3.31	0.007	1.78	1.07–2.96	0.026	1.77	1.06–2.94	0.028
Played in selection (team) (no = ref)	0.45	0.35–0.58	0.000	0.53	0.38–0.74	0	0.57	0.40–0.79	0.001	0.59	0.42–0.82	0.002
*Time use factors*												
Time use indicators *at this moment* (average hrs per week)												
Tennis/football outside sports club				0.27	0.22–0.34	0	0.27	0.22–0.34	0	0.28	0.22–0.34	0
Other sports				1.22	1.15–1.29	0	1.22	1.15–1.29	0	1.22	1.15–1.29	0
Other (fixed) hobbies				1.03	0.97–1.10	0.375	1.03	0.97–1.10	0.326	1.04	0.97–1.11	0.277
School/study and homework				1.00	0.99–1.01	0.756	1.00	0.99–1.01	0.726	1.00	0.99–1.01	0.84
(Part-time) job				1.02	1.00–1.03	0.135	1.02	1.00–1.04	0.108	1.02	1.00–1.04	0.12
Social activities *(no of times)*				1.16	1.04–1.31	0.011	1.16	1.03–1.31	0.013	1.17	1.04–1.32	0.01
Time spent on side activities at sports club *past season* (hrs)				0.97	0.94–0.99	0.014	0.97	0.94–1.00	0.019	0.97	0.94–1.00	0.028
Changes in job/school situation *since past season*												
Got job/increased work hrs (no = ref)				0.45	0.29–0.72	0.001	0.47	0.29–0.74	0.001	0.48	0.30–0.76	0.002
Changed schools/further educ. (no = ref)				2.96	2.04–4.28	0	2.97	2.04–4.31	0	2.92	2.01–4.26	0
*Environmental factors*												
Travel distance to sports club (km)							0.97	0.94–0.99	0.007	0.96	0.94–0.99	0.006
Neighbourhood density (strong – extremely urbanized = ref)									0.684			0.72
Rural							0.94	0.63–1.40	0.761	0.98	0.65–1.46	0.901
Hardly – moderately urbanized							1.10	0.77–1.57	0.599	1.12	0.78–1.60	0.544
*Motivational factors*												
Task orientation										0.84	0.71–0.99	0.032
Ego orientation										0.90	0.77–1.05	0.166
-2 Log likelihood	1595.78			1082.24			1068.95			1062.54		
Cox&Snell R^2^	0.05			8.49			0.39			0.93		
Nagelkerke R^2^	0.073			0.51			0.52			0.52		

## Discussion

This paper adds to the existing literature on dropout from youth sports by examining the extent to which factors related to time use and changes in time use, and the physical environment (e.g., travel distance and neighbourhood density) were associated with the probability of adolescents’ dropping out from football and tennis clubs.

First, the results indicate that determinants of dropping out from organized sports differed between young football and tennis members. For instance, time use characteristics affected adolescents’ dropout from football differently than it did for tennis. However, for both types of sports, time use determinants were more important predictors of dropping out than the environmental determinants of distance to the sports club and neighbourhood density. Change of schools (mainly the transition from high school to higher education) was by far the most important predictor of dropout from tennis, whereas this factor was not significantly associated with dropout from football. This is probably caused by the difference in time use and activity patterns of adolescents involved in football and tennis. For instance, tennis players spent significantly more time in school or on study than football players, whereas football players significantly spent more time on (part-time) jobs. Not surprisingly, tennis players had a higher education level than football players. Contrary to our expectation, starting a job or an increase in working hours decreased the odds of dropping out of tennis compared to those whose job situation remained the same, whereas this factor was of no significance in determining dropout from football. However, the total amount of time spent on jobs was not significantly associated with dropout from both sports. Furthermore, time spent on other sports was an important determinant of dropout from tennis. In contrast to football players, many young tennis players participate in one or more sports besides tennis. However, adolescents probably quit tennis as their ‘secondary’ type of sports because of time constraints and an increase of other responsibilities (such as, studying, working) and change in interests [15]. In addition, tennis players, as well as football players who spent more time practicing their sports outside the sports club were less likely to drop out. Apparently, their preference to practice their sports in an unorganized way, with flexible times, locations, and people [16], translates into a lower dropout probability. Another explanation might be that youths who are also outside the sports club being active practicing their sports might have a higher level of (task oriented) motivation and involvement, and are therefore less likely to quit their membership. Interestingly, time spent on social activities outside the sports club (tennis), or in social or other voluntary activities at the sports club (tennis and football), showed a negative association with dropout. The effect of time spent on social activities and other voluntary activities at the sports club, is of interest for sports federations and sports clubs who want to maintain their members and prevent youngsters from dropping out. Participating in voluntary and social activities at the sports club increases social connectedness, feeling of involvement and social capital [52, 53].

Our results indicate that environmental factors were the least important for dropping out compared to individual and time use factors, especially in football. The only significant effect was that members of tennis clubs who travelled larger distances to their tennis club were *less* likely to dropout. These results corresponded to the findings of Boiché & Sarrazin [48] who found that adolescents who continued with sports participation travelled more than those who dropped out. Youths with longer travel distances who continue to play tennis might be more motivated and/or are more task oriented to play, or might play at a higher competitive level (which may be associated with a higher ego orientation [18]), which requires more travel time to selection trainings and competitions. The relatively small travel distances by former football players correspond to the relatively great density and spread of football clubs across the country, and the moderate effect that travel distance has as a barrier for sports participation in the Netherlands [54]. A rather dense sports infrastructure in the Netherlands might also explain why neighbourhood density was not significantly associated with dropout.

In accordance with previous research [18, 21], we found that intrapersonal factors were important in explaining the probability of dropping out. Particularly, sex played an important role in predicting dropping out from football: girls aged 13–21 dropped out more frequently from football than boys. This seems striking, because football is increasingly popular among girls and numbers of female’s football clubs members have been growing in the Netherlands over the last decade [55]. Probably, there is also a group of girls who decides to quit their membership rather quickly, more so than boys. However, due to the low response rate among the football dropped outs and the overrepresentation of female dropped outs, this result has to be interpreted carefully. For tennis players, time use factors appeared to be more important predictors of dropout than sex, as the significant effect of sex disappeared when time use and environmental factors were added to the model. Sex differences in dropout determinants apparently can be explained by differences in time use between boys and girls. Furthermore, football and tennis players who had ever played in a selection team (as an indication of a higher competitive level) were less likely to drop out. This might be explained by a higher level of ego orientation and competiveness to play and practice sports, whereby dropout is less likely, as suggested by Balish et al. [18]. Finally, our results confirm the important role motivation plays for continuing participating in organized sports, as shown in previous studies [18, 22, 27]. Contrary to our expectations based on AGT and existing literature [27, 31, 32], we found that both task and ego orientations were associated with a lower probability of dropout, whether or not the sports itself was more team (football) or individual (tennis) orientated. Adolescents, who want to continue participating in organized sports and to actively engage within their sports teams and sports clubs, may benefit from having both task (wanting to learn and/or improve themselves) and ego (wanting to compete and win) orientations. In other words, having a high ego orientation does not need to have negative consequences for performance [56] and (continuing) sports participation [33]. It may however, be related to a higher level of competitiveness [18].

### Strengths and limitations of this study and future directions

A strength of this paper is the relatively large number of respondents in our total sample (*N* = 2555), compared to other studies on dropout from youth sports (with a range of *N* = 12 to *N* = 2180) [18]. Furthermore, our sample and study design allowed us to compare between members and former members of the two most important organized sports in the Netherlands. Despite the large number of respondents, the total mean response rate of this study was relatively low (6.4%). This may be related to our retrospective cross-sectional study design, whereas the majority of studies on dropout used prospective study designs in which participants were followed for a certain time period (on average 20.6 months) [18]. Especially the football dropouts showed a low response rate (3.5%) with a relatively overrepresentation of female dropouts and results regarding this subsample should therefore be interpreted carefully. Probably, the response rate among dropouts in general was lower because youths who already quitted their sports club membership felt less involved with the sport anymore and were therefore less likely to fill in the questionnaire while women were more likely to fill in. Furthermore, the differences in dropout determinants between football and tennis indicated that each (organized) sport has its unique sport specific characteristics and attracts different youth. It is likely that dropout determinants may differ for each type of organized sport in general, which has consequences for the generalizability of results of this study.

From a health perspective, we recommend that future research distinguish between youth who drop out of sports completely, and those who continue to participate in another type of (organized or flexible) sports. Moreover, determinants of dropout could be linked to actual motivations young people have for quitting their sports. Longitudinal research is needed in gaining insight into the sports participation behaviour of young people after dropping out of sports, and the causality of the relationships between time use and environmental characteristics, and participation in (different types of) sports.

## Conclusions

Although time use has been mostly neglected in studies focusing on adolescent’s dropout from sports, this study showed that time spent on activities, as well as important changes related to the school and job situations of adolescents were important predictors of dropout. However, determinants of dropout from sports differed to a great extent between football and tennis. Football and tennis probably attract different types of youth, with different interests, needs, and preferences for activities in time and space. In addition, differences in social context and organization of sports clubs may account for the different effects found. Furthermore, change of schools, as well as the time spent on another type of sports, increased the odds of dropout from tennis. Interestingly, time spent on social and voluntary activities at the sports club showed a positive association with continued membership of football and tennis clubs. Furthermore, longer travel distances between home and the tennis club decreased the probability of dropout from tennis. Similar to previous studies, intrapersonal factors, such as socio-demographic factors, education and motivational orientation (both task and ego orientated) showed significant associations with the probability of dropping out.

### Implications

Based on the findings from this study, we recommend to take time use variables into consideration as determinants of sports participation and dropout in socio-ecological models. Moreover, recommendations to sports and health professionals to keep young people involved in organized football and tennis include:

1. Offer more flexibility in schedules of training and competitions, to make it easier to accommodate sports club activities and competitions with other obligations and interests of youth.
2. Stimulate participation in social activities, and voluntary work at the sports club as it enhances continuing sports participation.
3. Pay special attention to prevent girls from dropping out. In this respect, future research could focus on how to further stimulate involvement with the sport and within the sports club, for instance by adjusting to the needs and interests of youths.
4. Encourage possibilities for youths to practice and play their sports outside of regular training hours, for instance at the sports club itself or at playgrounds or parks in the neighbourhood, as particating in these ‘free’ sports activities prevent from dropout.
